# Open three-stage transthoracic oesophagectomy versus minimally invasive thoraco-laparoscopic oesophagectomy for oesophageal cancer: protocol for a multicentre prospective, open and parallel, randomised controlled trial

**DOI:** 10.1136/bmjopen-2015-008328

**Published:** 2015-11-17

**Authors:** Juwei Mu, Shugeng Gao, Yousheng Mao, Qi Xue, Zuyang Yuan, Ning Li, Kai Su, Kun Yang, Fang Lv, Bin Qiu, Deruo Liu, Keneng Chen, Hui Li, Tiansheng Yan, Yongtao Han, Ming Du, Rongyu Xu, Zhaoke Wen, Wenxiang Wang, Mingxin Shi, Quan Xu, Shun Xu, Jie He

**Affiliations:** 1 Department of Thoracic Surgery, Cancer Hospital of Chinese Academy of Medical Science, Beijing, China; 2 Department of Thoracic Surgery, China-Japan Friendship Hospital, Beijing, China; 3 Department of Thoracic Surgery, Peking University Cancer Hospital, Beijing, China; 4 Department of Thoracic Surgery, Beijing Chao-Yang Hospital, Beijing, China; 5 Department of Thoracic Surgery, Peking University Third Hospital, Beijing, China; 6 Department of Thoracic Surgery, The Sichuan Province Cancer Hospital, Sichuan, China; 7 Department of Thoracic Surgery, The First Affiliated Hospital of Chongqing Medical University, Chongqing, China; 8 Department of Thoracic Surgery, Quanzhou First Hospital, Fujian, China; 9 Department of Thoracic Surgery, The People's Hospital Of Guangxi Zhuang Autonomous Region, Guangxi, China; 10 Department of Thoracic Surgery, Hunan Province Cancer Hospital, Hunan, China; 11 Department of Thoracic Surgery, Nantong Cancer Hospital, Jiangsu, China; 12 Department of Thoracic Surgery, Jiangxi Province People's Hospital, Jiangxi, China; 13 Department of Thoracic Surgery, The First Hospital of China Medical University, Liaoning, China

## Abstract

**Introduction:**

Oesophageal cancer is the eighth most common cause of cancer worldwide. In 2009 in China, the incidence and death rate of oesophageal cancer was 22.14 per 100 000 person-years and 16.77 per 100 000 person-years, respectively, the highest in the world. Minimally invasive oesophagectomy (MIO) was introduced into clinical practice with the aim of reducing the morbidity rate. The mechanisms of MIO may lie in minimising the reaction to surgical injury and inflammation. There are some randomised trials regarding minimally invasive versus open oesophagectomy, with 100–850 subjects enrolled. To date, no large randomised controlled trial comparing minimally invasive versus open oesophagectomy has been reported in China, where squamous cell carcinoma predominated over adenocarcinoma of the oesophagus.

**Methods and analysis:**

This is a 3 year multicentre, prospective, randomised, open and parallel controlled trial, which aims to compare the effectiveness of minimally invasive thoraco-laparoscopic oesophagectomy to open three-stage transthoracic oesophagectomy for resectable oesophageal cancer. Group A patients receive MIO which involves thoracoscopic oesophagectomy and laparoscopic gastric mobilisation with cervical anastomosis. Group B patients receive the open three-stage transthoracic oesophagectomy which involves a right thoracotomy and laparotomy with cervical anastomosis. Primary endpoints include respiratory complications within 30 days after operation. The secondary endpoints include other postoperative complications, influences on pulmonary function, intraoperative data including blood loss, operative time, the number and location of lymph nodes dissected, and mortality in hospital, the length of hospital stay, total expenses in hospital, mortality within 30 days, survival rate after 2 years, postoperative pain, and health-related quality of life (HRQoL). Three hundred and twenty-four patients in each group will be needed and a total of 648 patients will finally be enrolled into the study.

**Ethics and dissemination:**

The study protocol has been approved by the Institutional Ethics Committees of all participating institutions. The findings of this trial will be disseminated to patients and through peer-reviewed publications and international presentations.

**Trial registration number:**

NCT02355249.

## Introduction

Oesophageal cancer is the eighth most common cause of cancer worldwide.^1^ It is reported that the incidence and death rate of oesophageal cancer in China is the highest in the world, with an incidence of 22.14 per 100 000 person-years and a death rate of 16.77 pre 100 000 person-years, according to statistics on the incidence and mortality rates for oesophageal cancer in China in 2009.^2^ Surgery is still the gold standard for the treatment of resectable oesophageal cancer.

However, oesophagectomy for oesophageal cancer is a complex procedure, with morbidity and mortality rates of 23–50% and 2–8%, respectively, in western countries,^3^ ^4^ and of 9–29% and 2–4%, respectively, in China.^5^ ^6^

Minimally invasive oesophagectomy (MIO), which aims to reduce the morbidity rate, was first introduced into clinical practice in 1992.^7^ The mechanisms of MIO may lie in minimising the reaction to surgical injury and inflammation.^8^ Reduced morbidity and mortality rates of 11–25% and 1–3%, respectively, have been reported by many surgeons, which are lower than previous rates using the traditional open approach.^9–13^

Apart from observational studies,^9–13^ two completed randomised controlled trials (RCTs) in the Netherlands have reported promising results for MIO.^14^ ^15^ In the Netherlands study,^14^ a reduction of pulmonary infection rate was noted in the MIO group compared with the open oesophagectomy group, and the number of lymph nodes harvested were comparable in both groups, with manifest good oncologic effect in the MIO group. In the TIME (Traditional Invasive vs. Minimally invasive Esophagectomy) trial, the majority of the patients underwent surgery in a three-stage procedure, the patients having adenocarcinoma and squamous cell carcinoma (SCC). Moreover the technical complications in this trial were the same in the two groups, following neoadjuvant therapy. However, multiple surgical procedures were used in the study, and the complication rate was higher than in previous reports.^9–14^ In the French study,^15^ Mariette *et al* found that the rate of pulmonary complication was significant lower in the MIO group than in the open oesophagectomy group. The Ivor-Lewis procedure was used in the MIRO trial (Open vs Laparoscopically-assisted Esophagectomy for Cancer: A Multicentric Phase III Prospective Randomized Controlled Trial); however, a benefit from using the Ivor-Lewis MIO in that study may not be generalised to the McKeown oesophagectomy.

There are several ongoing randomised trials regarding the comparison of minimally invasive versus open oesophagectomy, with enrolment of over 100–850 subjects.^16–19^ The ROMIO (Randomized Oesophagectomy: Minimally Invasive or Open) trial is a three-arm trial which aims to compare the outcomes of total MIO versus hybrid MIO versus conventional open oesophagectomy (open thoracotomy and laparotomy).^16^ The procedures used in the ROMIO study include the open oesophagectomy or the MIO Ivor-Lewis procedure. The other three ongoing RCTs used the McKeown MIO procedure.^17–19^ The ROBOT trial was designed to compare the outcomes of robot-assisted McKeown MIO versus open McKeown oesophagectomy for resectable oesophageal cancer.^17^ Robot-assisted MIO has become popular in developing and developed countries in recent years.^20^ ^21^ However, it has not been as widely used as thoraco-laparoscopic MIO.

NCT02017002 is a trial which aims to compare the outcomes of the Ivor-Lewis and tri-incision approaches for patients with oesophageal cancer in Taiwan.^18^ The NCT02188615 trial is investigating the outcomes of neo-adjuvant chemoradiotherapy followed by MIO for squamous cell oesophageal cancer (NACRFMIE) in Taizhou China.^19^ The protocol used in the NCT02188615 study was the McKeown MIO with or without neo-adjuvant chemoradiotherapy. Although guidelines are supportive of neo-adjuvant chemoradiotherapy plus surgery over surgery alone,^22^ the reported studies lacked well-designed series, almost all mixing stages and types of tumour.^23^ Therefore, surgeons and oncologists might have different opinions about which modality to recommend, especially in clinical stage II or III.

Although the TIME and MIRO trials reported advantages of MIO over open oesophagectomy, currently the majority of oesophageal surgery is done by means of the open approach.^23^ Therefore, more studies are needed to clarify the role of MIO in the surgical treatment of oesophageal cancer. Here, we aim to conduct a multicentre, prospective, randomised, open controlled trial in order to evaluate the effectiveness of MIO versus open oesophagectomy through a McKeown procedure for the surgical treatment of resectable oesophageal cancer. We hope the results of our study will provide a high level of clinical evidence to support the routine use of MIO.

## Methods and analysis

This is a 3 year multicentre, prospective, randomised, open and parallel controlled trial, which aims to compare the effectiveness of minimally invasive thoraco-laparoscopic oesophagectomy to open three-stage transthoracic oesophagectomy for resectable oesophageal cancer.

Patients with resectable thoracic oesophageal carcinoma in cT1b-4aN0-2M0 are eligible for inclusion using chest CT, ultrasonography of the abdomen, head CT, and bone scan.^24^ We do not include a positron emission tomography (PET)/CT scan as a preoperative workup because medical insurance does not cover the expense of PET/CT. Cervical oesophageal cancer and adenocarcinoma of the oesophagogastric junction are excluded. In China, cervical oesophageal cancer is treated mainly with radiotherapy, and cancer of the oesophagogastric junction is resected via a single left thoracic approach.

**Figure 1:**
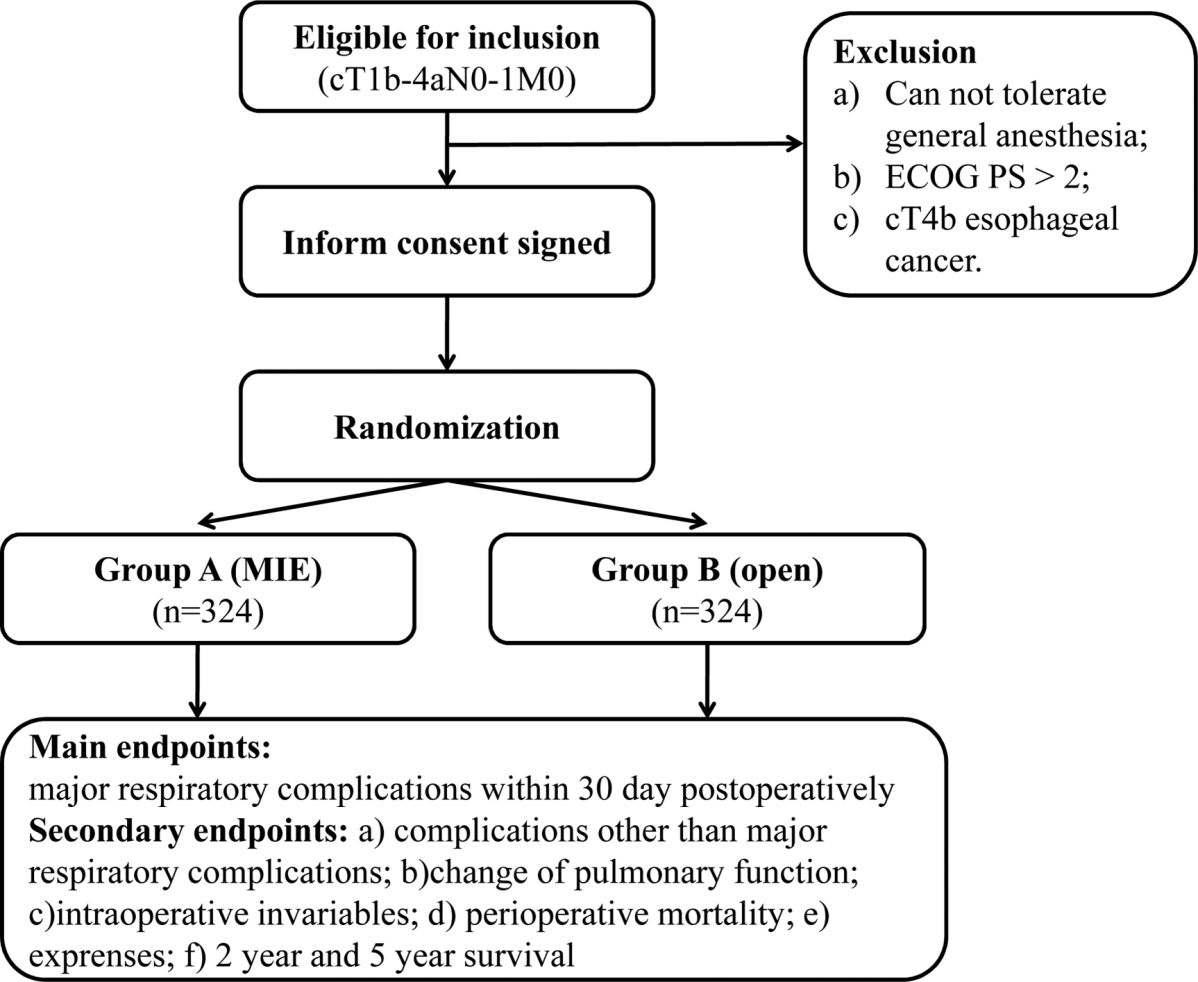
Flow chart of the study. ECOG PS, Eastern Cooperative Oncology Group Performance Status; MIE, minimally invasive oesophagectomy.

The patients are divided into two groups. Group A patients receive McKeown MIO which involves thoracoscopic oesophagectomy and laparoscopic gastric mobilisation with cervical anastomosis. Group B patients receive open McKeown oesophagectomy, which involves a right thoracotomy and laparotomy with cervical anastomosis. All patients received two field lymphadenectomy which involves resection of the lymph nodes in the thorax and abdomen. The flow chart for the trial is shown in figure 1. Neo-adjuvant chemotherapy will be performed for patients according to local guidelines of the participating cancer hospital.

### Objectives

The primary endpoints are major respiratory complications within 30 days after surgery. These respiratory complications involve respiratory distress or failure after the operation with continuation of mechanical ventilation, pulmonary atelectasis requiring sputum suction by bronchoscopy, pneumonia requiring specific antibiotics confirmed by thoracic X-ray or CT scan of the thorax and a positive sputum culture, and acute respiratory distress syndrome.

The secondary endpoints include other postoperative complications not involved in the primary endpoints according to systematic classification of morbidity and mortality after thoracic surgery.^25^ Other secondary endpoints include change of pulmonary function preoperatively and 3 months postoperatively, intraoperative variables involving volume of blood loss, duration of operation, the number and location of lymph nodes dissected, postoperative pain scale evaluated by pain score and quality of life questionnaires (EORTC QLQ-C30 and QLQ-0ES18), in-hospital mortality and 30-day mortality rate, the length of hospital stay, total expenses in hospital, 2 year survival rate, and survival at 5 years. The laboratory data include values for C-reactive protein and interleukin-6 from blood samples tested in the third and seventh day postoperatively in order to analyse the effect of MIO on surgery-related inflammatory reaction in the patients postoperatively.

### Participating surgeons and hospitals

All operations in the study are to be performed by surgeons with sufficient experience and skill in both open three-stage transthoracic oesophagectomy and minimally invasive thoraco-laparoscopic oesophagectomy. A surgeon who accomplished 30 cases of MIO annually was determined to be sufficiently experienced and skilled for our study. In order to prevent institution bias, only high-volume hospitals (>30 cases of MIO annually) will participate in the study.

Thirteen Chinese academic centres or hospitals will participate in the trial: Cancer Hospital of Chinese Academy of Medical Sciences, Beijing, China; Sino-Japan Friendship Hospital, Beijing, China; Beijing Cancer Hospital & School of Oncology, Peking University, Beijing, China; Chaoyang Hospital, Capital Medical of University; Peking University Third Hospital, Beijing, China; Sichuan Cancer Hospital, Sichuan, China; The First Affiliated Hospital of Chongqing Medical University, Chongqing, China; The First Hospital of Quanzhou City, Fujian, China; The People's Hospital of Guangxi Autonomous Region, Guangxi Autonomous Region, China; Hunan Cancer Hospital, Hunan, China; Nantong Tumor Hospital, Jiangsu, China; Jiangxi People's Hospital, Jiangxi, China; The First Hospital of China Medical University, Liaoning, China.

### Inclusion criteria

Subjects may enter the trial with all of the following: (1) oesophageal carcinoma confirmed by pathology; (2) resectable thoracic oesophageal carcinoma in cT1b-4aN0-2M0 using chest CT preoperatively, ultrasonography of the abdomen, head CT and bone scan; (3) oesophageal carcinoma that can be resected initially by multidisciplinary treatment, or that can be resected after neoadjuvant therapy; (4) age between 18 and 75 years; (5) Eastern Cooperative Oncology Group Performance Status (ECOG PS) score ≤2; (6) a life expectancy ≥12 months; (7) tolerate tracheal intubation and general anaesthesia as determined by an anaesthetist preoperatively; (8) laboratory findings including liver and kidney function, and electrolyte findings in 14 days before operation meet the criteria; (9) informed consents must be signed before the beginning of any procedures in the study.

### Exclusion criteria

Subjects may not enter the trial with one of the following: (1) cervical oesophageal cancer and adenocarcinoma of the oesophagogastric junction; (2) history of thoracic or abdominal operations which may affect the study; (3) unable to tolerate tracheal intubation and general anaesthesia as determined by an anaesthetist preoperatively; (4) severe comorbidities such as any unstable systemic disease, including active infection, uncontrolled hypertension, angina within previous 3 months, congestive heart failure, myocardial infarction within previous 6 months, severe arrhythmias, and liver, kidney or other metabolic diseases; (5) poor compliance of follow-up; (6) pregnant or lactating women; (7) ECOG PS scores >2; (8) other patients considered unsuitable such as those who do not agree to participate in the trial.

### Ethics

The trial is conducted in accordance with the principles of the Declaration of Helsinki and the International Conference on Harmonisation Good Clinical Practice (ICH-GCP), local laws and regulations. The study protocol has been approved by the institutional ethics committees of all participating institutions. During the study, all modifications, extensions and updates of trial procedures should be reviewed and approved by the medical ethics committee in every participating centre.

### Randomisation

When the eligible patients are confirmed and informed consent is obtained, the researchers login through the trial randomisation system and input the patient's number and other related information. Then the patient is randomised to either the open three-stage transthoracic oesophagectomy group or the minimally invasive thoraco-laparoscopic oesophagectomy group through a group number produced by SPSS software.

### Trial intervention (surgical technique)

#### Minimally invasive thoraco-laparoscopic oesophagectomy

##### Thoracoscopic phase

Minimally invasive thoraco-laparoscopic oesophagectomy has been described previously.^13^ The patient is placed in the left lateral decubitus position. The position of the double-lumen tube is verified, and single-lung ventilation used. Four thoracoscopic ports are established. A 10 mm port is placed at the seventh intercostal space, just along the anterior axillary line, for the camera. Another 10 mm port is placed at the eighth or ninth intercostal space, posterior to the axillary line, for the dissection instrument (ultrasonic coagulating shears) and passage of the end-to-end circular stapler (EEA; Covidien or Johnson) or Hem-lock. A 5 mm port is placed in the anterior axillary line, at the third or fourth intercostal space, and this is used to pass a fan-shaped retractor to retract the lung anteriorly and allow exposure of the oesophagus. A 5 mm port is placed just below the subscapular tip to place the instruments for retraction and counter traction. The inferior pulmonary ligament is divided. The mediastinal pleura overlying the oesophagus is divided and opened to the level of the azygous vein to expose the thoracic oesophagus. The azygous vein is then dissected and divided with an endoscopic vascular stapler or Hem-lock. The thoracic oesophagus, alone with the peri-oesophageal tissue and mediastinal lymph nodes, is circumferentially mobilised from the diaphragm to the level of inlet of the thorax. Mediastinal lymphadenectomy is undertaken for every patient including the region of left recurrent and right subclavian, paratracheal, subcarinal, left and right bronchial, lower posterior mediastinum, para-aortic, and para-oesophageal lymph nodes. Following the procedure the chest is inspected closely, and haemostasis verified. A chest tube is routinely placed.

##### Laparoscopic phase

The patient is placed in a supine position. A pneumoperitoneum (12–14 cm H_2_O) is established by carbon dioxide injection through an umbilical port. A total of five abdominal ports (three 5 mm and two 40 mm) are used. After placement of the ports, the first step of the laparoscopic phase involves exploration of the abdomen to rule out advanced disease. The mobilisation of the stomach is initiated with division of the greater curvature using a Harmonic scalpel (Ethicon Endo-Surgery, Ohio, USA). The short gastric vessels are divided with ultrasonic coagulating shears. The gastrocolic omentum is then divided, with care taken to preserve the right gastroepiploic artery. The posterior attachments of the stomach are then divided after retraction of the stomach anteriorly. The left gastric vessel is divided at its origin from the coeliac trunk with an endoscopic gastrointestinal anastomosis stapler or Hem-lock. Lymphatic tissues around the vessels are included in the resection. Subsequently, the right crus is visualised and dissected, followed by dissection and definition of the left crura of the diaphragm. The abdominal/distal oesophagus is dissected as far as possible toward the distal end. The gastric conduit is made extracorporeally. Pyloroplasty or gastric drainage procedure are not routinely performed in our study, and a feeding jejunostomy tube created is not created. Instead, we insert a duodenal nutrition tube before the anastomosis, as follows: using sterile gloves, a candy ball is enclosed, peeled and fixed to the front end of the feeding tube through the small laparotomy incision; the feeding tube is then pushed until the front end and the candy ball lie in the duodenum, and the rest of the feeding tube is placed into the gastral cavity and bound with the nasogastric tube; then, the nasogastric tube is pulled out from the nose and fixed; and the nasogastric tube is then reinserted into the gastric cavity. The abdomen is inspected to make sure that haemostasis is adequate and the incisions are closed.

#### Cervical anastomosis

After the laparoscopic phase and the thoracoscopic phase, a 4–6 cm horizontal neck incision is made. The cervical oesophagus is exposed. Careful dissection is performed down until the thoracic dissection plane is encountered, generally quite easily since the video-assisted thoracoscopic surgery (VATS) dissection is continued well into the thoracic inlet. The oesophagogastric specimen is pulled out of the neck incision and the cervical oesophagus divided high. The specimen is removed from the field. An anastomosis is performed between the cervical oesophagus and the gastric tube using standard techniques (mechanical stapled or hand sewn anastomosis in an end-to-side fashion).

#### Open three-stage transthoracic oesophagectomy

As in the minimally invasive thoraco-laparoscopic oesophagectomy, a three-stage procedure is followed in the open group. The first stage is started with a right posterolateral thoracotomy. The mediastinal pleura overlying the oesophagus is divided with an electrotome. The thoracic oesophagus, along with the peri-oesophageal tissue and mediastinal lymph nodes, are circumferentially mobilised from the diaphragm to the level of inlet of the thorax. The second stage is the mobilisation of the stomach which is initiated with the division of the greater curvature using ultrasonic coagulating shears. The short gastric vessels are divided with ultrasonic coagulating shears as well. The gastrocolic omentum is then divided, with care taken to preserve the right gastroepiploic artery. The posterior attachments of the stomach are then divided after retraction of the stomach anteriorly. The left gastric vessel is divided at its origin from the coeliac trunk with sutures. Lymphatic tissues around the vessels are included in the resection. Subsequently, the abdominal oesophagus is dissected as far as possible toward the distal end. Pyloroplasty is not routinely performed. The abdomen is inspected to make sure that haemostasis is adequate and the incisions are closed. For the last stage, the cervical incision is made and then the anastomosis is performed like for MIO.

### Postoperative care

The patients are placed in intensive care units or discharged to hospital wards directly from the operating theatre according to the guidelines of the participating centre. Assessment of recurrent laryngeal nerve injury is undertaken on the first day postoperatively. Postoperative respiratory tract management includes chest physiotherapy and early ambulation. Patient-controlled analgesia is given to every patient to control postoperative pain.

### Sample size calculation

According to the literature, the incidence of respiratory complications after oesophagectomy for oesophageal carcinoma is 27–31%.^2^ ^3^ Therefore, we plan to decrease the incidence rate of respiratory complications from 30% to 20% with minimally invasive thoraco-laparoscopic oesophagectomy. This is based on a unilateral significance level of α=0.025 and a power of β=0.8. After adding 10% loss of the sample, 324 patients will be required for each group so a total of 648 patients will finally be enrolled into the study.

### Statistical analysis

Statistical analyses are carried out using SPSS software for Windows, V.16.0 (SPSS Inc, Chicago, Illinois, USA). Continuous variables are presented as mean±SD and compared using Student's t test or analysis of variance (ANOVA) test. Categorical variables will be reported as absolute numbers (frequency percentages) and analysed using χ^2^ test. Survival will be estimated by means of Kaplan-Meier curves, and survival compared using log-rank test. A two-tailed p<0.05 is considered statistically significant.

## Discussion

Although adenocarcinoma of the oesophagus has become the main type of oesophageal cancer in western countries, oesophageal SCC is still the predominant histologic type in China. Therefore, both Ivor-Lewis and McKeown oesophagectomy are important in the surgical treatment of oesophageal SCC. The TIME and MIRO trials concluded that MIO is not only feasible, but perhaps superior to open oesophagectomy. However, there are no RCTs designed to compare the outcome of the MIO McKeown procedure and the open McKeown procedure for oesophageal SCC, apart from one study which aims to compare the outcomes of McKeown MIO with or without neo-adjuvant chemoradiotherapy (NCT02188615) for squamous cell oesophageal cancer. Therefore, we are conducting this study, which aims to investigate the difference between the MIO McKeown procedure and the open McKeown procedure for oesophageal SCC.

Maas *et al*^8^ found that less surgical trauma could lead to better preserved acute-phase and stress responses and fewer clinical manifestations of respiratory infections in patients who underwent MIO compared to those who underwent open oesophagectomy. Our previous study showed that the overall morbidity rate was significantly decreased in the MIO McKeown group compared with the open McKeown group, and no significant differences were found in the number of harvested lymph nodes.^13^ For these reasons, we hypothesise that the MIO McKeown procedure may result in a significant decrease in major respiratory complications compared with the open McKeown procedure for oesophageal SCC, without comprising the oncologic clearance.

This is the largest multicentre, prospective, RCT designed to compare open McKeown oesophagectomy with MIO McKeown oesophagectomy for oesophageal cancer in China. We hope the results of this study will add new evidence to support the use of MIO in the surgical treatment of oesophageal cancer.
